# 1444. Pediatric Tuberculosis in Mexican Children: A Retrospective Analysis of 100 Patients

**DOI:** 10.1093/ofid/ofac492.1272

**Published:** 2022-12-15

**Authors:** Enrique G Villarreal, Ricardo J Estrada-Mendizábal, Emilia Ramos-Barrera, Pablo D Treviño-Valdez, Oscar Tamez-Rivera

**Affiliations:** Tecnologico de Monterrey, Escuela de Medicina y Ciencias de la Salud, San Pedro Garza Garcia, Nuevo Leon, Mexico; Tecnologico de Monterrey, Escuela de Medicina y Ciencias de la Salud, San Pedro Garza Garcia, Nuevo Leon, Mexico; Tecnologico de Monterrey, Escuela de Medicina y Ciencias de la Salud, San Pedro Garza Garcia, Nuevo Leon, Mexico; Tecnologico de Monterrey, Escuela de Medicina y Ciencias de la Salud, San Pedro Garza Garcia, Nuevo Leon, Mexico; Tecnologico de Monterrey, Escuela de Medicina y Ciencias de la Salud, San Pedro Garza Garcia, Nuevo Leon, Mexico

## Abstract

**Background:**

Tuberculosis (TB) is a disease caused by *Mycobacterium tuberculosis* complex, mainly by *M. tuberculosis.* Annually, 1 million children develop TB disease and many more present a latent form of infection. The objective of this study is to define the epidemiological trends of the different clinical presentations of TB in pediatric population.

**Methods:**

A retrospective, cross-sectional study, carried out at a pediatric referral hospital in Mexico between 2012-2021. Pediatric patients diagnosed with TB were included. Descriptive statistics were performed in order to summarize the demographic, clinical and paraclinical characteristics of pediatric patients with TB. A Chi-squared univariate analysis was performed evaluate the association between several variables of interest with mortality.

Diagnostic criteria for TB in children according to the Mexican Clinical Practice Guidelines

**Figure:**
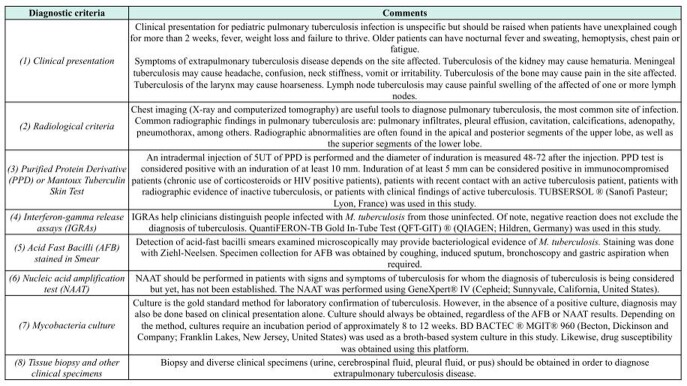

**Results:**

A total of 100 patients were included in the study. Pulmonary TB was the most common presentation (51%), followed by ganglionic (21%), meningeal (14%), and milliary (7%). A male predominance was observed (54%). Mean age of presentation was 7.8 years ± 1.5, most lived in urban areas (79%) and had a positive COMBE (57%). Half of the patients had BCG vaccination. Fifty-two patients were successfully cured, 36 patients were receiving antituberculous treatment and fatality rate was 12%. Meningeal TB was the most fatal clinical presentation. Fever was the most common sign (65%), followed by cough (56%) and weight loss (37%). The most common sign of meningeal TB were generalized seizures (10/14), fever (10/14), and vomiting (7/14). Brain imaging was performed in all patients with meningeal TB: hydrocephalus (71.4%) and basal arachnoiditis (64.3%) were present. Cerebrospinal fluid analysis was also performed: 11 patients had pleocytosis, 10 had hyperproteinorrhachia, and 10 had hypoglycorrhachia. Clinical TB presentation (p=0.009) and immunodeficiency (p= 0.015) were significantly associated with mortality.

Epidemiological Characteristics According to TB Clinical Presentation.

**Figure:**
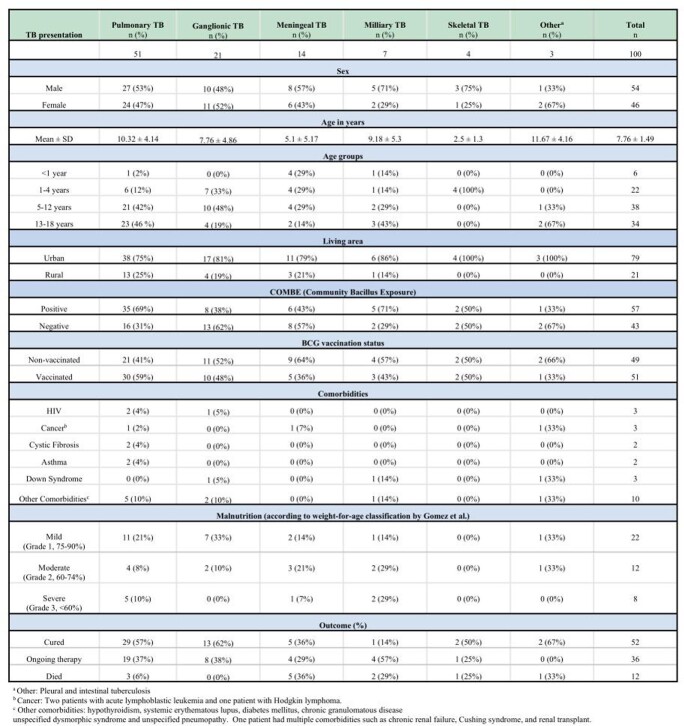

Signs and symptoms in children according to their clinical presentation of TB.

**Figure:**
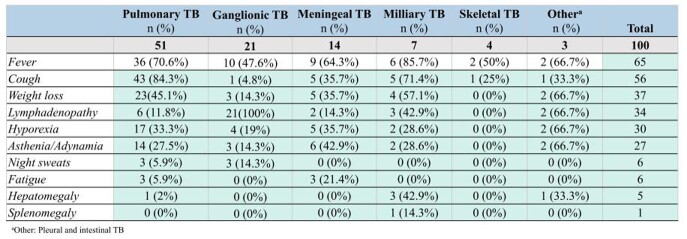

Clinical, radiological and cerebrospinal fluid characteristics in meningeal TB.

**Figure:**
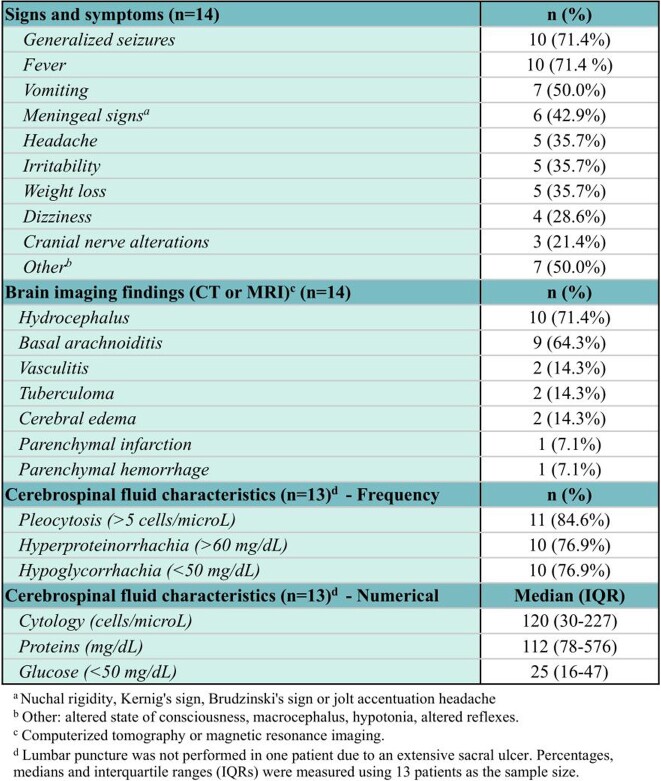

**Conclusion:**

Pediatric TB cases in this referral hospital indicated an equivalent proportion of pulmonary and extrapulmonary TB. Most of the patients with milliary and meningeal TB were not vaccinated; however, BCG vaccination was not significantly associated with mortality. Patients with extrapulmonary forms of the disease and with immunodeficiency were associated with mortality.

Clinical outcomes of children with TB according to their epidemiological characteristics

**Figure:**
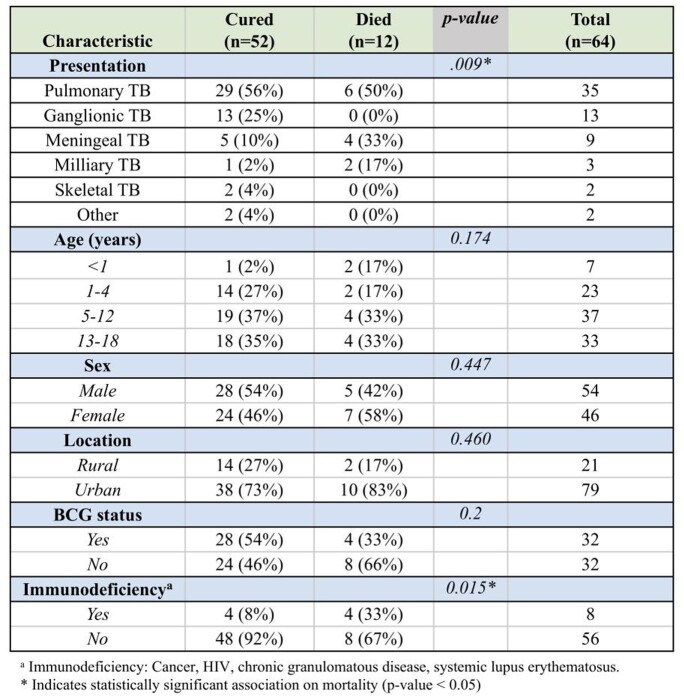

**Disclosures:**

**All Authors**: No reported disclosures.

